# Supplementary material for: Causal Relationship between Aging and Anorexia Nervosa: A White-Matter-Microstructure-Mediated Mendelian Randomization Analysis
Source: Biomedicines. 2024 Aug 16;12(8):1874. doi: 10.3390/biomedicines12081874 (PMC11351342; doi:10.3390/biomedicines12081874)
Supplement: Supplementary file 1 [file biomedicines-12-01874-s001.zip › Supplementary Figure/Figure S8/Leaveoneout plot of MR analysis for TL to ubm-b-1611.pdf]

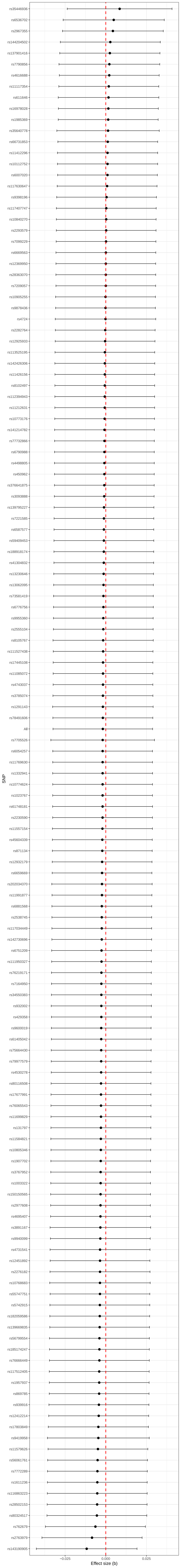

rs35446936
rs6536702
rs2967355
rs144204502
rs137901416
rs7790856
rs4616688
rs11117354
rs611646
rs16978028
rs1985369
rs35640778
rs66731853
rs11412296
rs10112752
rs6007020
rs117630647
rs9398196
rs117407747
rs10840270
rs2293579
rs7099229
rs6669563
rs12369950
rs28363070
rs7209057
rs10905255
rs9878436
rs4724
rs2282764
rs12925933
rs113525195
rs142426306
rs11426156
rs8102497
rs112394943
rs11212631
rs10773176
rs141214782
rs77732866
rs6790988
rs4498805
rs450962
rs376641875
rs3093888
rs139795227
rs7221585
rs6587577
rs59409453
rs188918174
rs41304832
rs13230646
rs13062095
rs73581419
rs6776756
rs9955360
rs2555104
rs8105767
rs111527438
rs17445108
rs11085072
rs4743037
rs3785074
rs1291143
rs78491606
All
rs7705526
rs6054257
rs11769630
rs1332941
rs10774624
rs1023767
rs61748181
rs2230590
rs11557154
rs45604339
rs871134
rs12932179
rs6659669
rs202034370
rs11991877
rs6881568
rs2538745
rs117034449
rs142730696
rs6751209
rs111950327
rs76219171
rs7164950
rs34550383
rs932002
rs429358
rs9600019
rs61405042
rs75664430
rs79977579
rs4530278
rs80116508
rs17677991
rs76065543
rs11699829
rs131797
rs11584821
rs10805346
rs1907702
rs3767952
rs1003322
rs150150565
rs2977608
rs4695407
rs3891167
rs9940099
rs4731541
rs12451892
rs2276182
rs10768683
rs55747751
rs5742915
rs182059586
rs139669835
rs56799554
rs185174247
rs76666449
rs117512405
rs1957937
rs869785
rs939916
rs12412214
rs17803849
rs9419958
rs11579626
rs56061761
rs7772289
rs1611236
rs116863223
rs28502153
rs80324517
rs762679
rs2763979
rs143190905
SNP
−0.025
0.000
0.025
Effect size (b)
